# Investigation of the mechanism of Buyang Huanwu decoction in improving learning and memory impairment in Alzheimer's disease mice based on lipidomics

**DOI:** 10.1007/s11418-025-01890-x

**Published:** 2025-04-07

**Authors:** Jing Jiang, Kai Duo, Siyu Zhu, Yitong Wang, Hui Xue, Chengyu Piao, Yifan Ren, Xia Lei, Yafeng Zhang, Jianxin Liu, Lihong Yang, Ning Zhang

**Affiliations:** 1 College of Pharmacy, Heilongjiang University of Chinese Medicine, Harbin, Heilongjiang, China; 2 Heilongjiang Institute for Drug Control, NMPA Key Laboratory for Quality Research and Evaluation of Traditional Chinese Medicine, Harbin, Heilongjiang, China; 3 Jiangsu CM Clinical Innovation Center of Degenerative Bone & Joint Disease, Wuxi TCM Hospital Affiliated to Nanjing University of Chinese Medicine, Wuxi, Jiangsu, China; 4 School of Pharmaceutical Sciences, China-Pakistan, International Science and Technology Innovation Cooperation Base for Ethnic Medicine Development in Hunan Province, Hunan University of Medicine, Huaihua, Hunan China

**Keywords:** Alzheimer's disease, Buyang Huanwu Decoction, Lipidomics, Calycosin-7-glucosidel, PPARγ

## Abstract

In this study, a lipid disorder Alzheimer’s disease (AD) model was developed with high-fat diet and d-galactose injected intraperitoneally (HFD & d-gal) to evaluate the activities of Buyang Huanwu Decoction (BYHWD) compared with donepezil hydrochloride. The learning and memory abilities of BYHWD were evaluated by Morris water maze test (MWM). The lipid levels in serum, histopathology, and immunohistochemistry of hyperphosphorylated tau protein in hippocampal neurons were conducted to prove the therapy effects of BYHWD. After the identification of constituents absorbed into the brain using LC–MS, UPLC-TQ-MS was employed to analyze endogenous lipid metabolites in the hippocampi of mice. Based on the validated differential markers identified through lipidomics analysis, we further substantiated potential therapeutic pathway of BYHWD through the application of molecular docking technology. The mechanism underlying BYHWD was subsequently confirmed by palmitic acid-injured HT22 cells. The results showed that BYHWD significantly improved the cognitive deficits and regulated the lipid levels of HFD & D-gal mice. BYHWD also protected the neuronal cell condition of hippocampal neurons, increased the density of dendritic spines, and reduced the expression of P-tau. Lipidomics revealed that 41 differential lipid metabolites were retuned after BYHWD administration, and this change may be related to the PPARγ pathway. Calycosin-7-glucoside showed good interaction with PPARγ in vivo composition analysis. Calycosin-7-glucoside increased the mRNA expression levels of lipid metabolism-related enzymes and PPARγ, as well as the expression of PPARγ protein in vitro study. BYHWD activated the PPARγ pathway to induce peroxisome proliferation and regulated lipid metabolism disorders in the AD mice brain.

**Graphical abstract:**

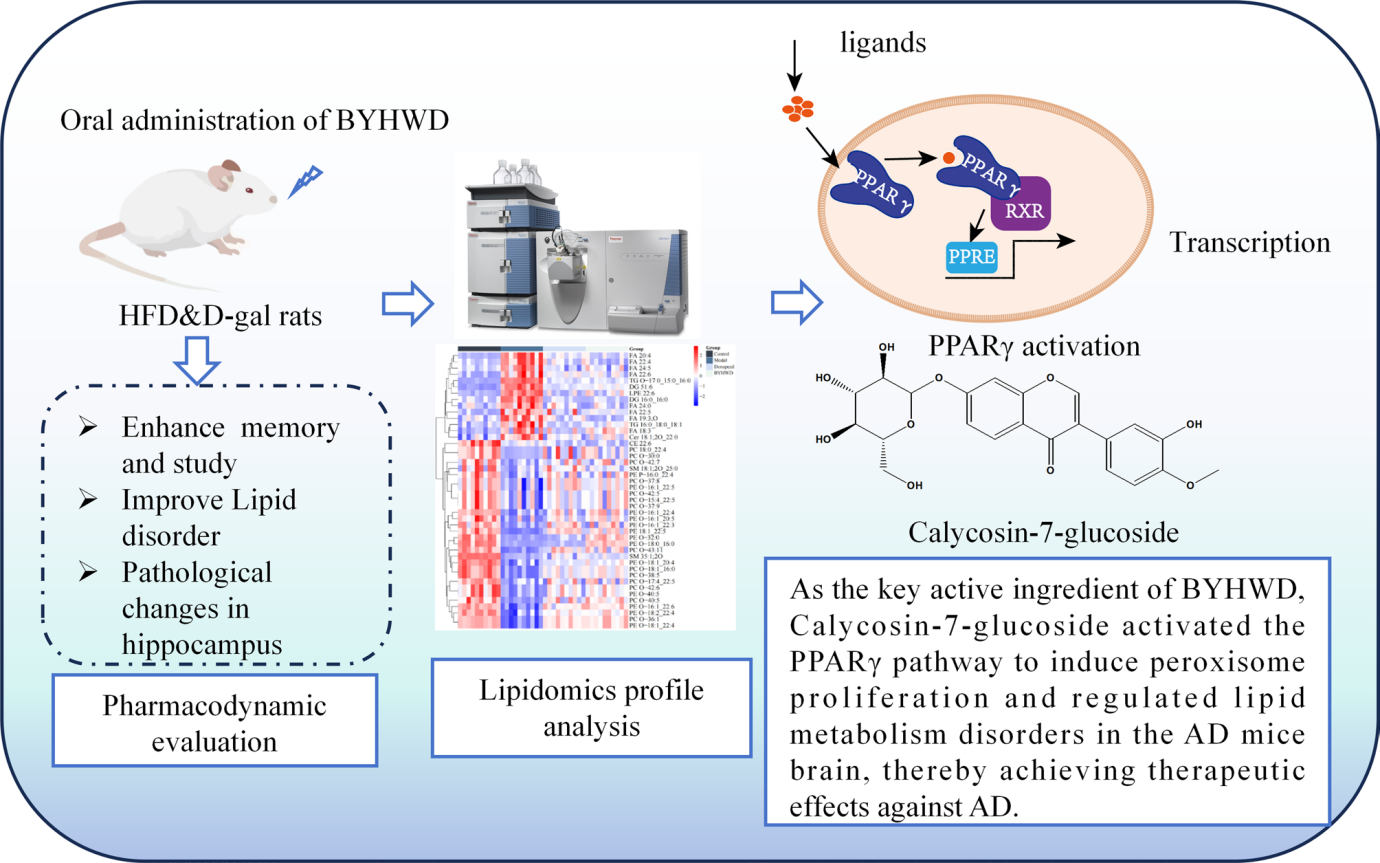

**Supplementary Information:**

The online version contains supplementary material available at 10.1007/s11418-025-01890-x.

## Introduction

Alzheimer's disease (AD) is a neurodegenerative disorder disease affecting the central nervous system, characterized by progressive cognitive impairment and behavioral deterioration [1]. The etiology of AD remains incompletely understood, and currently there are no efficacious therapeutic interventions or pharmacological treatments available to cure or effectively manage its onset and progression [2].

Extensive literature has reported dyslipidemia as a significant acquired risk factor for AD, with elevated cholesterol levels increasing the susceptibility to AD development [3]. Animal studies have demonstrated the presence of AD markers in animals fed with a high-cholesterol diet, while statins, commonly employed to reduce blood cholesterol levels, exhibit potential preventive effects against AD [4]. Furthermore, AD patients revealed lipid metabolism disorders characterized by excessive accumulation of long-chain fatty acids and elevated ceramide levels in the brain, along with diminished peroxisome count and decreased expression of PPARγ protein and mRNA. These results indicated that disrupted brain lipid homeostasis was a main reason of AD [5].

The Buyang Huanwu decoction (BYHWD), originating from Wang Qingren's monograph Yi Lin Gai Cuo during the Qing Dynasty. According to the Chinese Pharmacopoeia (2005), the formula includes the following 7 herbs: ① Radix Astragali (*huangqi*), the dried roots of Astragalus membranaceus (Fisch.) Bge.var. mongholicus (Bge.) Hsiao; ② the carda part of Radix Angelicae Sinensis root (guiwei), the dried lateral roots of *Angelica sinensis* (Oliv.) Diels; ③ Radix Paeoniae Rubra *(chishao*), the dried roots of *Paeonia lactiflora* Pall.; ④ Rhizoma Chuanxiong (*chuanxiong*), the dried rhizomes of *Ligusticum chuanxiong* Hort; ⑤ Flos Carthami (*honghua*), the dried flowers of *Carthamus tinctorius* L.; ⑥ Semen Persicae (*taoren*), the dried seeds of *Amygdalus persica* L.; and ⑦ Pheretima (*dilong*), the dried bodies of *Pheretima aspergillum* (E. Perrier), in the ratio of 120∶6∶4.5∶3∶3∶3∶3 on a dry weight basis, respectively. [6, 7]. Recently, animal experiments have demonstrated that BYHWD effectively ameliorated cognitive impairment in AD model mice. Furthermore, BYHWD was reported to therapy AD with an impressive treatment effective by reducing pathological Aβ precipitation, alleviating neuroinflammatory response, improving blood–brain barrier permeability, improving mitochondrial function, reducing reactive oxygen species and reducing oxidative stress. It is remarkable that BYHWD exhibited the ability to regulate disrupted lipid metabolism both in experimental animals and patients, leading to a significant increase in high-density lipoprotein (HDL-C) levels while reducing low-density lipoprotein (LDL-C), triglyceride (TG), and total cholesterol (TC) levels [8]. BYHWD was confirmed to reduce the blood lipid concentration in atherosclerosis rats by inhibiting the NF-κB signaling pathway and modulate the absorption, transportation, and metabolism of lipids by regulating the expression of enzymes and transporters in lipid metabolism [9, 10].

Since few studies exploring the anti-AD effects of BYHWD via the perspective of brain lipid metabolism, this study focused on the exploration the main effective constituents and active pathway of BYHWD on lipid metabolism regulation for treatment of AD. This study employed HFD & D-gal mice as AD models to investigate the impact of BYHWD on enhancing cognitive function and ameliorating lipid metabolic disorders. Based on the data of lipidomics with LC–MS, we further investigated the underlying mechanisms involved in correcting lipid metabolic disorders and exerting neuroprotective effects, thereby establishing a pharmacological basis for our study with palmitic acid-induced HT22 cells.

## Materials and methods

### Chemicals and reagents

D-galactose (D-gal) was provided by Solarbio Science & Technology Co., Ltd. (Beijing, China). Donepezil hydrochloride tablets were obtained from Eisai Pharmaceutical Co. Ltd. (Shanghai, China). Toluidine blue staining kit was provided by Sangon Biotech (Shanghai) Co., Ltd. Mayer's hematoxylin stain solution was obtained from Shanghai Yuanye Bio-Technology Co., Ltd. (Shanghai, China). Hito CryoMyelinStain™ Nissl kit and Hito Golgi-Cox OptimStain™ kit were obtained from Beijing Biolead International Trading Co., Ltd. (Beijing, China). Anti-P-tau antibody and anti-PPAR gamma antibody were obtained from Jiangsu Pro-Tech Biological Research Center Co. (Changzhou, China). Enhanced chimiluminescence kit was purchased from Beyotime Biotechnology (Shanghai, China). Palmitic acid and calycosin-7-glucoside (CG) were purchased from Sigma-Aldrich LLC. (Shanghai, China). GW9662 and Oil Red O staining kits were purchased from Biyuntian Biotechnology Institute (Shanghai, China). β-Actin monoclonal antibody and HRP-labeled sheep anti-rabbit IgG were purchased from Beijing Zhongsui Jinqiao Biotech Co. (Beijing, China).

### Preparation of BYHWD

The BYHWD herbs were supplied by the First Affiliated Hospital of Heilongjiang University of Traditional Chinese Medicine, with authentication by Professor Sun Huifeng. the dried roots of *Astragalus membranaceus (huangqi*), the dried lateral roots of *Angelica sinensis* (*dang gui*), the dried roots of *Paeonia lactiflora* (*chishao*), the dried bodies of *Pheretima aspergillum* (*dilong*), the dried rhizomes of *Ligusticum chuanxiong* (*chuanxiong*), the dried flowers of *Carthamus tinctorius* (*honghua*), the dried seeds of *Amygdalus persica* (*taoren*) at the ratio of 120:6:4.5:3:3:3:3 were ground and extracted by reflux heating for 2 h with tenfold water twice. The combined filtrate was concentrated to 4.5 g/mL (calculated with herbs) for use.

### Experiment animals and administration

3-month-old SPF male Kunming mice were purchased from Department of Laboratory Animals, Harbin Medical University (SCXK (hei) 2024–002). The mice were accommodated in a condition of constant cycles of 12-h light and 12-h dark at room temperature (21 ± 2.5 °C and 50 ± 10% humidity), with free access to standard rodent chow and water. All animal studies were performed in accordance with institutional guidelines for the care and use of laboratory animals, and the protocol was approved by the Animal Research Ethics Committee of Heilongjiang University of Chinese Medicine, and were also approved by it (No. 2024042823).

**Fig. 1:**
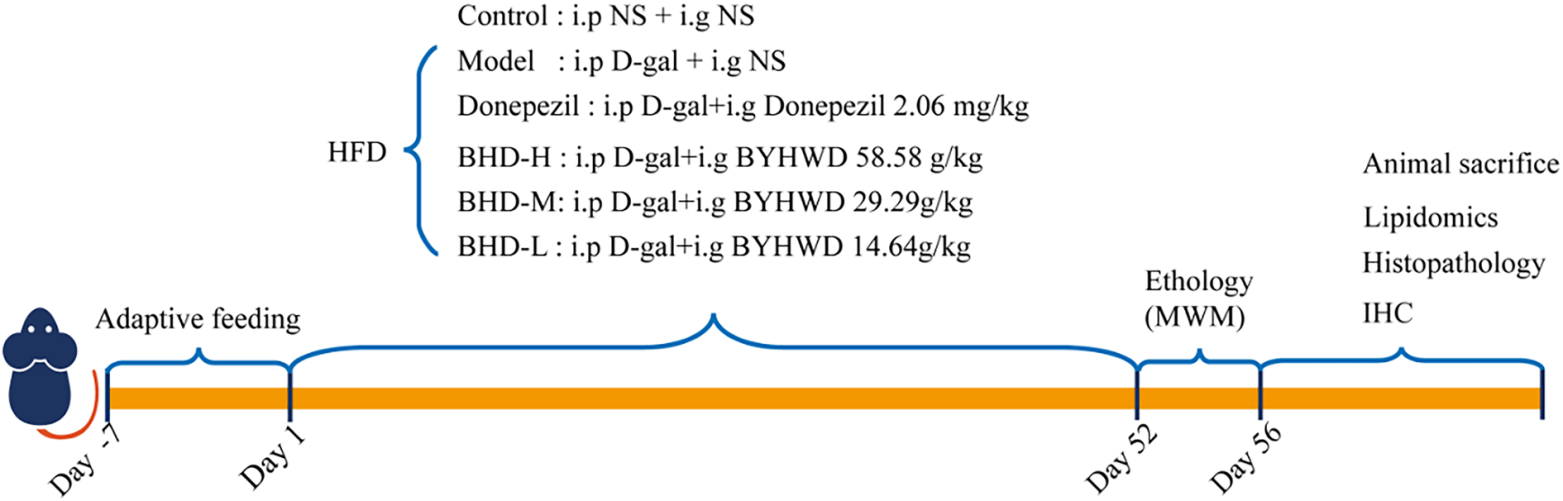
Schematic diagram of modeling process

The AD model mice were replicated by injection of D-gal intraperitoneally and fed with high-fat diet (HFD, 45% fat, Product Number, 1,016,712,400,381,763,584, Keao Xieli Feed Co., LTD, Beijing, China) for 56 consecutive days. The AD model mice were orally administered with high dosed BYHWD at 58.58 g/kg/d calculated with herbs (BHD-H group), middle-dosed BYHWD at 29.29 g/kg/d (BHD-M group), low-dosed BYHWD at 14.64 g/kg/d (BHD-L group) from day 1 to day 52. Donepezil at the dose of 2.06mg/kg/d (donepezil group) was used as positive control. In steadily, mice in control group were injected intraperitoneally with saline and orally administered saline for 52 consecutive days. Each group consisted of 15 mice, resulting in a total of 90 mice used. From day 52 to day 56, behavioral tests were conduct. Mice of each group were sacrificed by the removal of the eyeballs to harvest blood then the brain tissue were collected on 56th day. The hippocampi were stripped immediately for lipidomics and histomorphology analysis. Serum was taken to analyze the lipid indicators using the automated biochemical analyzer (Toshiba 40, Japan). The experimental process was shown in Fig. 1.

### Morris water maze (MWM)

On the 52nd day of treatment, all of the mice were subjected to the Morris water maze test in the accordance with the protocol. The orientation navigation test was applied from the 52nd day to the 55th day. Morris water maze behavior test was used to evaluate the learning and memory ability of mice. We conducted the MWM test according to the previous protocol, described with modifications [11–15]. Briefly, the mice were placed in rooms 24 h in advance to acclimatize before starting the experiment. We used a circular water tank with a diameter of 120 cm and a height of 50 cm. A movable platform with a diameter of 4.5 cm and a height of 30 cm was set in the second quadrant of the water tank. The platform was 2 cm below the water surface and the water temperature was maintained at (23 ± 1 °C). All mice were trained for 4 days, 4 times a day (60 s/ time, 30 min interval). If the animals couldn’t find the platform within 90 s, they were placed on the platform for 30s. On the 5th day, the platform was removed and the mice were allowed to swim freely for 90 min, The time that the mice stayed in the platform quadrant and the number that the mice crossed the platform position within 90 s were recorded.

### Serum HDL-C, LDL-C, TG and TC measurement

Concentrations of high-density lipid cholesterol (HDL-C), low-density lipid cholesterol (LDL-C), triglyceride (TG), and total cholesterol (TC) in collected mice serum were determined by automated biochemical analyzer (Toshiba 40, Japan).

### Histomorphology

#### Hematoxylin & eosin (H&E) staining

Hippocampal neurons were observed using H&E staining. The mice brain tissues were fixed in a 4% paraformaldehyde solution for over 24 h at a temperature of 4 ℃, followed by dehydration using a series of gradient alcohols and xylenes. The samples were immersed and embedded in wax cylinders. The paraffin-embedded samples were sectioned into 5-μm slices and subsequently subjected to Hematoxylin & eosin staining using the standard protocol of the kit in order to observe the pathological alterations occurring in the hippocampal region. Images were captured and analyzed under a light microscope (IX71; Olympus, Japan) at a magnification of × 200.

#### Nissl staining

Nissl staining was involved to observe the morphological and quantitative changes of Nissl bodies. The wax slices were subjected to the H&E gradient ethanol dehydration procedure, followed by immersion in a toluidine blue solution for 5 min and staining in a constant temperature oven for 40 min. Subsequently, the wax slices were rinsed with water and dehydrated using the same method as employed in H&E staining.

#### Golgi staining

Golgi staining was used to observe synaptic morphology and dendritic spine density. Following the kit instruction manual, brain tissue was immersed in a daily prepared mixture of solutions A and B for at least 14 days. Then the A and B solutions were replaced by solution C and the tissue was immersed for another 3 days. The immersed tissue was washed with water and dehydrated by gradient ethanol and transparently treated with xylene solutions I and II, followed with paraffin embedding. The hippocampal area was observed by a microscope at a × 100 field of view.

### Immunohistochemistry

Immunohistochemistry assay for the expression of P-tau. H&E staining sections were incubated in 3% H_2_O_2_ for 10 min at room temperature, rinsed in water, and soaked in PBS for 5 min. Then the sections were placed in citrate buffer and repaired by microwave heating. The serum was discarded after incubation for 20 min with dropwise serum closure. Anti-Phospho-tau (Ser202/Thr205) antibody (1:200 dilution, Jiangsu Pro-Tech Biological Research Center Co. Changzhou, China) and HRP-labeled sheep anti-rabbit IgG (1:200 dilution, Beijing Zhongsui Jinqiao Biotech Co. Beijing, China) were added sequentially. The positive proteins stained with diaminobenzidine and hematoxylin were subjected to analysis.

### Lipidomics

#### Lipidomics sample preparation

The lipid metabolome analysis utilized hippocampal tissue obtained from animal experiments 20 mg mice hippocampus tissue was homogenized with 400 μLsaline and then was vortexed with methyl tert-butyl ether for 1 min. 50 μL of isopropanol/acetonitrile/water solution (v:v:v = 75:20:5) and 750 μL methanol were added into 50 μL hippocampus extract [16]. The mixture was vortexed for 2 min to extract lipid and remove protein. Then 2.5mL of methyl tert-butyl ether was added and vortexed for 10 min, followed by 10 min vortex of 0.625 mL water. After 10 min centrifuge (13,000 rpm) at 4° C, the upper layer was collected and evaporated under nitrogen. The residue was resolved with 200 μL of isopropanol/acetonitrile/water solution (v:v:v = 75:20:5). After centrifuge at 13,000 rpm for 10 min at 4° C, the upper layer was analyzed with LC–MS.

#### Chromatographic and mass parameters

Lipidomic analysis was performed on SCIEX ExionLC AD HPLC (AB sciex Co., USA), coupled with a Triple TOF 5600 MS system. The lipids separation was achieved on an ACQUITYUPLCHSS T3 (100 mm × 2.1 mm, 1.8 μm) column maintained at 55°C with a flow rate of 0.40 ml/min. The gradient elution was consisted of mobile phase A (10 mM ammonium formate and 90% isopropanol/acetonitrile) and mobile phase B (10 mM ammonium formate and 60% acetonitrile/water) under gradient elution condition. The gradient elution program was 0–2 min, 5% B; 2–2.1 min, 5%–15% B; 2.1–12 min, 30% B; 12–22 min, 30%–70% B; 22–22.1 min, 70% B; 22.1–25 min, 5% B. 5 μl of each sample was injected to analyze. The scanning ranges for positive and negative ionization detection modes in primary and secondary mass spectrometry were 100–1800 m/z and 50–1800 m/z respectively. The nitrogen gas was used to each gas path. The ion spray voltage was set at + 5500 v and -4500 v, the curtain gas pressure was maintained at 35 psi, and both ion source gasses were regulated to 60 psi, the ion source temperature was controlled at 550 ℃. The declustering potentials for primary and secondary mass spectrometry were set at 80 v and 90 v respectively. The collision energy was adjusted to 10 v for primary mass spectrometry and increased to 35 v for secondary mass spectrometry.

### Analysis of the constituents of BYHWD in vivo

Twelve AD model mice were respectively orally administered with BYHWD. The dosage and the method of molding and administration were conducted as described in Sect. "Experiment animals and administration". After 1 h of intragastric administration, the mice were sacrificed and the hippocampus tissues were removed immediately. Then, 4 times of homogenate solution (0.2% formic acid–water 1:4) was added to homogenate for 10 min. 0.4 mL cold 0.2% formic acid–acetonitrile solution was quickly added into 0.2 mL homogenized mixture, swirled and mixed well, then was centrifuged at 4 ℃ and 12,000 rpm for 5 min. The supernatant was ready for analysis.

The analysis was performed with SCIEX ExionLC AD HPLC (AB sciex Co., USA), coupled with a Triple TOF 5600 MS system. The sample separation was achieved on an ACQUITYUPLCHSS T3 (100 mm × 2.1 mm, 1.8 μm) column maintained at 40°C with a flow rate of 0.30 ml/min. The linear gradient elution of water containing 0.1% formic acid and acetonitrile. The gradient elution program has been designed to 0–2 min, 5% B; 2–6 min, 5–30% B; 6–7 min, 30% B; 7–10 min, 30–60% B; 10–11.5 min, 60% B; 11.5–13 min, 60–80% B; 13–13.5 min, 80% B; 13.5–16 min, 80%–90% B; 16–17 min, 90% B; 17–17.5 min, 90–100%B; 17.5–20 min, 100% B; 20–20.5 min, 100%–5% B; 20.5–23 min, 5% B. The scanning ranges for positive and negative ionization detection were 100–1200 m/z and 50–1200 m/z respectively. The ion spray voltage was set at + 5500 v and − 4500 v, the curtain gas pressure was maintained at 35 psi, and both ion source gasses were regulated to 50 psi; the ion source temperature was controlled at 550 ℃. The declustering potentials for primary and secondary mass spectrometry were set at 90 v, respectively. The collision energy was adjusted to 10 v for primary mass spectrometry and increased to 40 v for secondary mass spectrometry.

#### Molecular docking

The binding capacity between active ingredients and key targets was validated through molecular docking. We have demonstrated the brain-entry of six components in D-gal model mice after oral administration of BYHWD. Calycosin-7-glucoside, Paeoniflorin, Baicalein, Formononetin, Astragaloside IV, Senkyunolide H. Donepezil and GW9662 were additionally evaluated through molecular docking studies using PPARγ as a control. The TCMSP database (https://old.tcmsp-e.com/tcmsp.php) was initially employed to retrieve the 2D structures of these six small molecule compounds, which were subsequently optimized into mol2 format files suitable for computer-based molecular simulations using 3Dchem software. Subsequently, the crystal structure of PPARγ was obtained from the PDB database (https://www.rcsb.org/) and dehydrated using pymol software. The Surflex-Dock GeomX high-precision module in SYBYL was ultimately employed for the precise molecular docking of small molecules.

#### Cell validation

HT22 cells induced by palmitic acid were used as a cellular model of AD, and the PPARγ antagonist GW9662 was used to investigate whether CG ameliorate lipid disorders and exert neuroprotective effects by activating the PPARγ pathway. HT22 cells (highly differentiated, No. CL0162) were purchased from Feng Hui Biotechnology Co (Changsha, China) and cultured in Dulbecco's Modified Eagle Medium (Gibco, USA) with 10% fetal bovine serum (ExCell Bio, Suzhou) and 1% penicillin–streptomycin solution, 100X, at 37 °C with 5% CO_2_. HT22 was stimulated with palmitic acid (400 μmol/L) for 2 h in the presence or absence of CG (1000 nmol/L) incubation for 24 h or GW9662 (20 μmol/L) incubation for 1 h. The supernatants and the cell lysates were collected and analyzed the lipid deposition, PPARγ protein expression, and mRNA levels of lipid metabolism-related enzymes using Oil Red O staining, RT-PCR, and western blotting.

#### Oil Red O staining

The medium was removed and cells were rinsed twice with PBS. The Oil Red O fixative was incubated for 25 min, followed by removal of the fixative and two subsequent washes with water. Then the cells were rinsed for 30 s by 60% isopropanol, dip-dyed in the configured Oil Red O staining solution for 60 min. Next the cells were rinsed for 30 s by 60% isopropanol and washed for 4 times by water. The hematoxylin staining solution was added for 1 min and washed for 4 times by water. After discarding the Oil Red buffer following a 1-min incubation, the cells were observed under a microscope.

#### Analysis of RT-PCR

The quantification of mRNA levels of enzymes was involved in lipid metabolism in cellular systems. Total RNA was extracted and reverse-transcribed into cDNA using the SGExcel Ultra SYBR Mixture (withROX) kit. The primer sequences were listed in Table 1. The reaction was conducted using a gradient PCR amplifier and an Mx3000P real-time fluorescence quantitative PCR instrument (TProfessional standard Biometra, Germany). The reaction mixture was composed of 1.8 μL forward primer (10 μmol/L), 1.8 μL reverse primer (10 μmol/L), 45 μL SGExcel UltraSYBR Mixure (withROX) × 2, and 37.8 μL RNase-Free ddH2O water. The reaction conditions were as follows: pre-denaturation at 95 ℃ for 3 min, denaturation at 95 ℃ for 15 s, annealing for 20 s, extension at 72 ℃ for 25 s, a total of 40 reaction cycles, and final extension at 72 ℃ for 10 min. The relative expression amount of mRNA was 2^−ΔΔCt^, of which Ct was the threshold period,$\Delta Ct = \overline{x}_{Ct1} - \overline{x}_{Ct2}$, Ctl was the investigatory period of the indicator primer, and Ct2 was the threshold period of β-actin.

**Table 1:** Primer sequence of RT-PCR assay

Primer name	Forward primer (5’–3’)	Reverse primer (3’–5’)
*AGPS*	ACCAGATTCCCTGGAGTTCA	GAACCACCAGGTCCTCGATA
*GNPFA*	TACAACTGGGTTCTGAAAGCC	CAGCTGCCAAAGATCGAAGT
*CHPT1*	TCCAGTTCTTGGATTTCTAGGTGGAGT	ACACTGGTGCCTGCTATAGTGGA
*ACOX1*	GAGGGGAACATCATCACAGG	AAAGTCAAAGGCATCCACCA
*Catalase*	CCTCGTTCAGGATGTGGTTT	TGCCTTGGAGTATCTGGTGA
*ABCA1*	GATCTTCCAAGCAGCCAAAG	TCAAACTTCCAGCCTCCTTC
*PPARγ*	CAGGAGCAGAGCAAAGAGGT	TGGACACCATACTTGAGCAGA
*β-actin*	ACCTTCTACAATGAGCTGCG	CTGGATGGCTACGTACATGG

#### Western blotting

The supernatants obtained from HT22 cells after treatment were subjected to precipitation. The cells were harvested using a cell lysis buffer, and the total protein concentration was determined through the BCA assay. Equal amounts of protein were loaded on SDS–polyacrylamide gel, transferred onto PVDF membranes and blocked in 5% skimmed milk for 2 h. Subsequently, PVDF membranes were incubated in the presence of the primary antibody against PPARγ overnight at 4 ℃ and followed by HRP-conjugated secondary antibody incubation for 2 h. The protein expression was visualized using an enhanced chemiluminescence kit and photographed by the Tanon 4600 automatic chemiluminescence image analysis system (Tanon Science and Technology Co., Ltd., Shanghai, China).

#### Statistical analysis

Statistical analysis was performed using SPSS 17.0 software (SPSS, Inc.). Multivariate analysis of repeated measures data was used to escape latency. One-way ANOVA was performed using post hoc LSD or Tamhane'sT2 based on normality and chi-squared variance, or using the Kruskal–Wallis nonparametric test. Image-Pro Plus 6.0 was used to image analysis and western blot analysis.

## Results

### Effect of BYHWD on ameliorating cognitive deficits and the regulation of serum lipid levels in HFD & D-gal-induced AD model mice

#### Morris water maze test

**Fig. 2:**
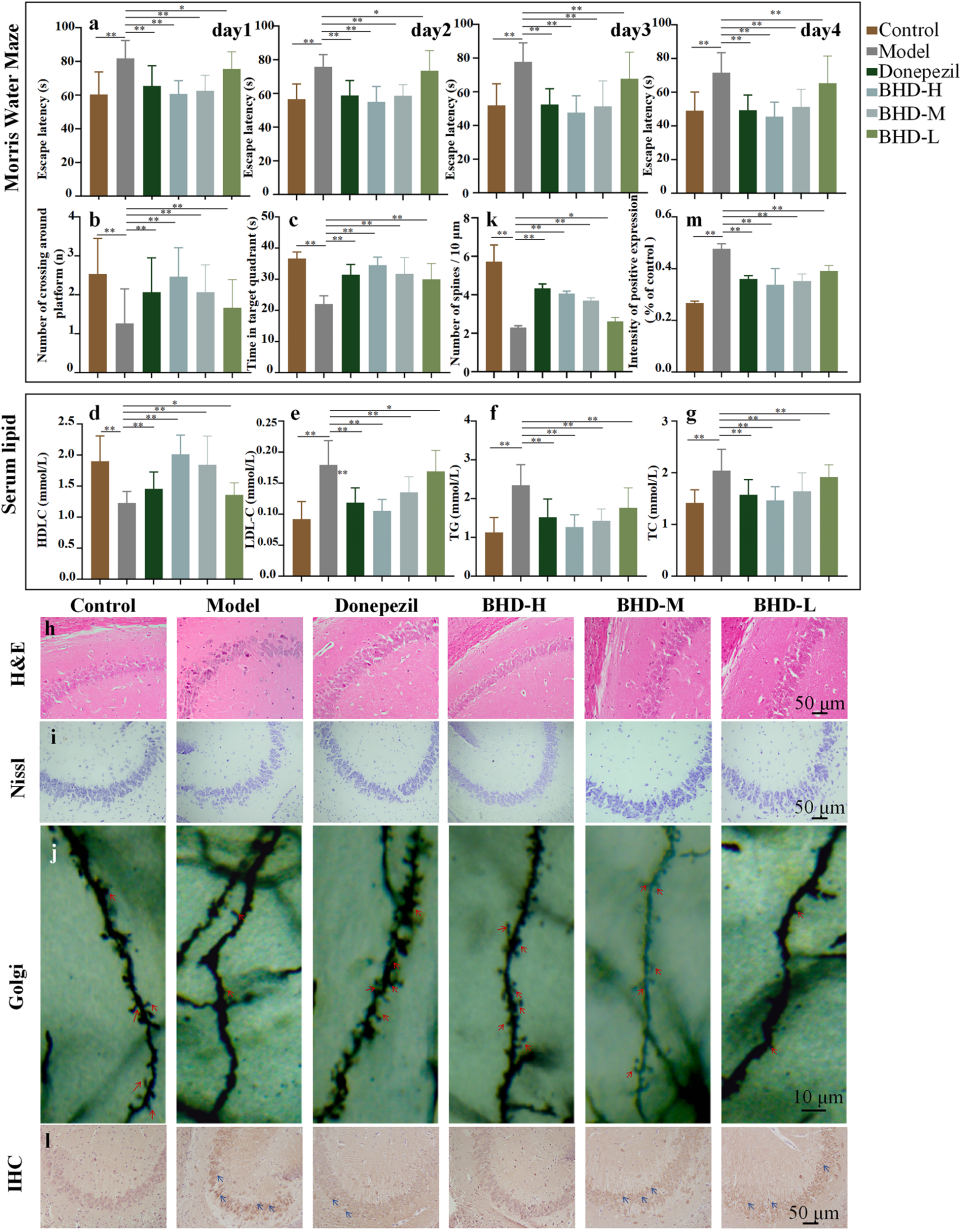
Effect of BYHWD on ameliorating cognitive deficits and the regulation of serum lipid levels in HFD & D-gal-induced AD model mice. The **a-c** are the results of the water maze test. **a **The escape latency during the training period; **b** The frequency of mice traversing the platform quadrant; **c** Average duration of mice' stay in the platform quadrant in MWM test (n = 15); The **d-g** are the effects of BYHWD on serum lipid.** d** HDL, **(e)** LDL, **(f)** TG,** (g)** TC (n = 6). The **h-m** are the results of pathological staining. (**h**) Morphology of neurons with H&E staining; **i** Nissl corpuscles with Nissl staining; (**j**) The density of dendritic spine with Golgi staining (×400 the red arrows are spines) in the hippocampus of mice; **(k)** Number of spines / 10μm. **(l)** Representative immunohistochemical stain (×200 the blue arrows are p-Tau positive cells) images of the p-Tau in the hippocampus of mice, **(m)** The area quantification in the hippocampus of mice in the positive particles of p-Tau, n = 3; Data are expressed as the mean ± SD, *p < 0.05, **p < 0.01 vs model group

MWM test was conducted to evaluate the neuroprotective effect of BYHWD on the learning and memory deficits in HFD & D-gal induced AD model mice (Fig. 2 a-c). In the probe test (without the platform), HFD & D-gal mice exhibited a reduced frequency of crossing the original position of the platform and spent less time in the target quadrant compared to normal mice, indicating the existence of impaired memory of HFD & D-gal AD model. The mice in the HFD & D-gal group exhibited no improvement in spatial learning ability, as evidenced by their inability to locate the hidden platform, when compared to the control group. Gradually decreasing escape latencies were observed in the donepezil group and BHD-H group over the during the place navigation period over four consecutive days. Both mice treated with donepezil or BHD-H showed significant improvements in spatial learning after training, as evidenced by increased crossings of the original location of the missing platform and prolonged stays in the target quadrant, especially on day 5. Notably, the BHD-H treated model mice had a 25% reduction in escape latency on day 4 compared with day 1. Although the BHD-M and BHD-L groups showed an improvement trend in the escape latency, the number of crossing platforms and the time in targeted quadrant, the effects were not as obvious as that of the BHD-H group. Together, these results indicated that BHD-H treatment could noticeably ameliorate the cognitive deficits of HFD & D-gal-induced AD model mice.

#### The regulation of serum lipid levels

Dyslipidemia is correlated with impairment of reduced cognitive function and other pathological symptoms of AD which is considered as one of the significant risk factors for AD [17, 18]. In this research, HDL-C, LDL-C, TG and TC levels in serum were measured to evaluate the lipid metabolism in mice. As the results shown in Fig. 2, the serum level of HDL-C, which was suppressed in the HFD & D-gal mice was remarkably restored by BHD-H. The increased LDLC, TG, and TC level induced by a HFD & D-gal were significantly attenuated by the administration of BHD-H. In comparison, the serum lipid was treated by BYHWD, BHD-H had the best ability to increased the levels of HDL-C and reduced LDL-C, TG, and TC (Fig. 2 d-g). The results showed that HFD & D-gal model could cause dyslipidemia in mice and BHD-H could improve it.

### Pathological changes of BYHWD in the hippocampus

In order to verify the pathological changes of HFD & D-gal model and BYHWD on hippocampus, HE staining, Nissl staining and Goggi staining were performed on the CA3 region of hippocampus. Nissellite can be used as a marker of the neurons functional, and it is abundant in the normal function neurons. When neurons are damaged, the nissellite quantity decreases, disintegrates and even disappears [19]. Dendritic spines are spines on the dendritic branches of neurons, and are the main sites for forming synapses between neurons. The number and the shape of dendritic spines are regarded as the basis of neuron function. The number of dendritic spines in the normal brain is large and the morphology is robust, while the number from model animal with abnormal cognitive is relatively rare and the morphology is comparatively slender [20]. Aiming at the investigation on whether the HFD & D-gal model could cause hyperphosphorylation of tau protein in the hippocampus of mice, a typical pathological manifestation of AD [21], and the therapeutic impact of BYHWD on this pathological manifestation, immunohistochemical staining was accomplished.

#### H&E staining

The cells in the hippocampal region of mice in the control group exhibited a well-organized arrangement, characterized by a substantial number of neurons, uniform staining, distinct nuclei, and absence of nuclear consolidation (Fig. 2 h). The hippocampal cells of mice in the model group showed disorganization, neuronal loss, cellular shrinkage, inconspicuous nuclei, and evident nuclear condensation, which indicated that the success of HFD & D-gal model. Similar to the BHD-H and BHD-M group, observations from the donepezil group revealed a more orderly distribution of neurons within the hippocampal region accompanied by increased neuron count as well as preserved morphology and clearer nuclei without obvious nuclear consolidation. The BHD-L group was slightly better than the model group but not as well as the high-dose groups. The neuroprotective effect of BHD-H on AD model mice was observed.

#### Nissl staining

The hippocampal neurons of the control group mice exhibited a tightly organized arrangement with regular morphology, abundant intracellular nidus, and deeply pigmented blue patches that were distinctly visible. As shown in Fig. 2i, compared with the control group, the hippocampal neurons in the model group of mice were loosely arranged and disordered, and the number of intracellular nidus was significantly reduced and lightly colored. Both the donepezil and BHD-H groups demonstrated abundant and densely packed neuronal intracellular nidus in the hippocampal region of mice, characterized by a significantly higher number and darker coloration. Treatment with donepezil and BHD-H effectively reduced neuronal cell death in mice. These results suggested that BHD-H had a potential protective effect on HFD & D-gal-induced mice neurons of hippocampal.

#### Golgi staining

Golgi staining was carried out to assess changes in dendrites and dendritic spines within hippocampal neurons. As shown in Fig. 2 j-k, the neuronal cytosolic dendrites appeared distinct and intact. In comparison to the control group, the model group displayed reduced dendritic spine density, unclear dendritic structure, and a significant decrease in number. Dendritic spine density was significantly higher in the donepezil and BHD-H groups compared to the model group. The density of dendritic spines in BHD-M group and BHD-L group was less than that in BHD-H group. The results of Golgi staining revealed that BHD-H treatment rescued dendritic spine deterioration of model mice.

#### Hippocampal immunohistochemical stain

Immunohistochemical detection was involved to assess the expression of phosphorylated tau protein (P-tau) in the mice hippocampus (Fig. 2 l-m). Compared with the control group, the expression of P-tau in mice of the model group was significantly increased (p < 0.01). Compared with the model group, the expression of P-tau protein in the donepezil and BHD-H group mice was significantly reduced (p < 0.01). In contrast, there was no significant change in BHD-M group and BHD-L group. The results verify that the AD model is successful and BHD-H could ameliorate tau protein hyperphosphorylation effectively in the hippocampus.

As the pharmacodynamic experiment results showed that the treatment effect of BHD-H group on AD mice was more significant than that of BHD-M and BHD-L, so the following studies were conducted with the dose of BHD-H.

### Lipidomics

#### Lipidomics profile analysis

**Fig. 3:**
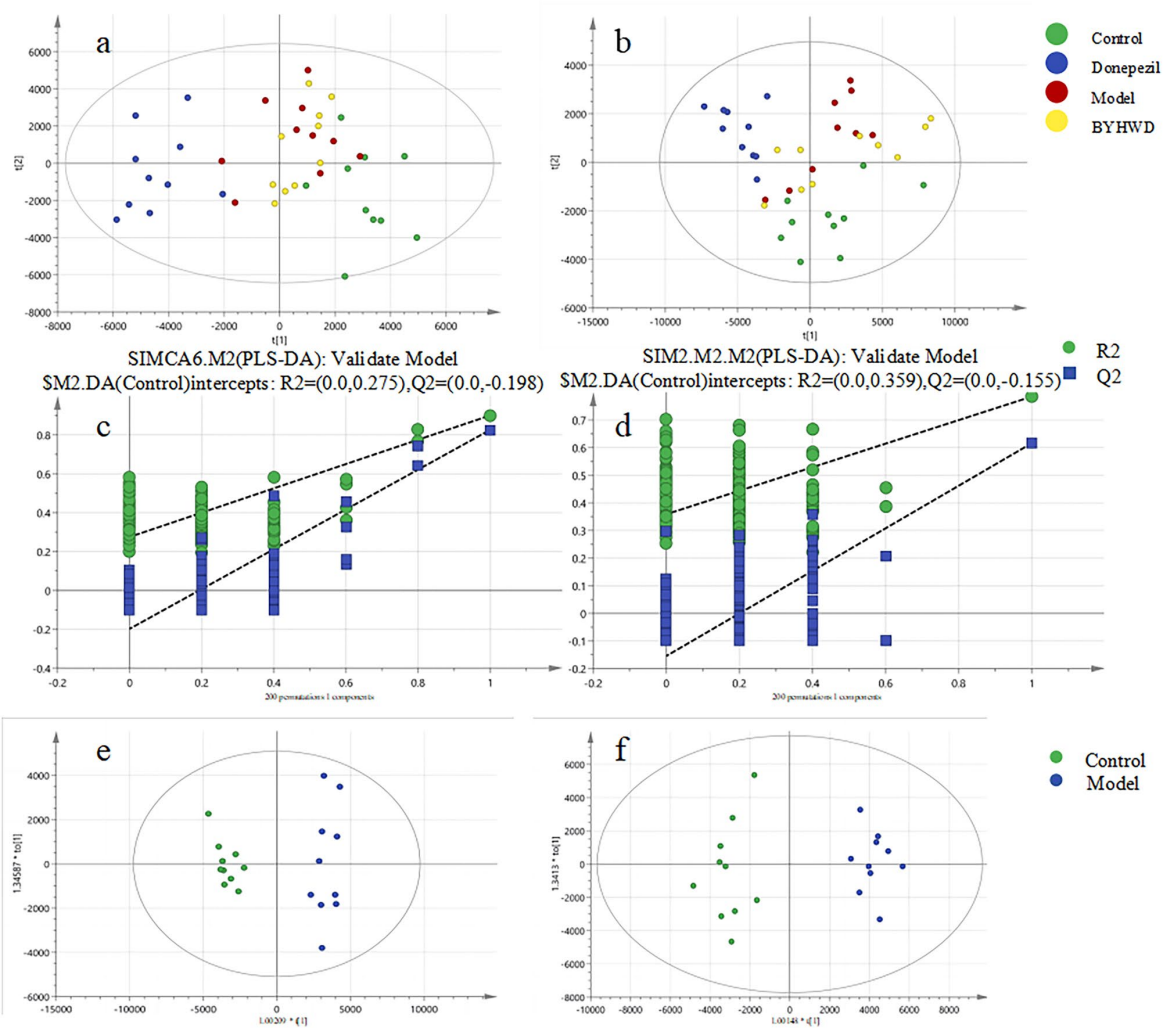
Alterations in lipid metabolite profiles within the hippocampal tissue of mice across different groups. The PLS-DA score graph in positive** a** and negative **b** ion mode. Permutations graph in positive **c** and negative** d** ion mode. The OPLS-DA score graphs in positive** e** and negative** f** ion mode

In order to investigate the mechanism underlying the therapeutic effects of BYHWD on AD, a lipidomics profile analysis was conducted on hippocampal tissues from mice. The positive and negative MS data of hippocampus from the mice in each group administrated intragastric in abf format were analyzed with MSDIAL and SIMCA-P software for data manipulation [22–24]. The total ions chromatogram of hippocampal tissue of mice in each group and the QC samples are shown in the supplementary material 1 Fig. S1–S2. The processed data were calculated and analyzed with partial least squares-discriminant analysis (PLS-DA) to explore the changes in lipid metabolism of mice hippocampal tissues. Each dot in different colors represented each sample in different groups. A permutation test was conducted to the PLS-DA model (n = 200) [25]. All data dots to the left of R2 and Q2 were consistently lower than those on the right, with a positive regression slope (Fig. 3 c-d). These findings indicated that the model exhibited excellent adaptability and predictive performance.

As shown in Fig. 3 a-b, the dots of the control, model and BYHWD group were clearly separated. It can be seen from the PLS-DA plot that the model group exhibited a different metabolic pattern with the control group, demonstrating that HFD & D-gal interfered with the normal metabolism of endogenous lipids in healthy mice. It was also found that the dots of BYHWD group were closer to those of control group, indicating that the lipidomics disorder in the model group was significantly regulated after gavage of BYHWD. The donepezil group also had a tendency to regulate, but the tendency was not as significant as that of BYHWD group.

A further analysis of orthogonal partial least squares-discriminant analysis (OPLS-DA) was used to analyze the metabolites above mentioned. In Fig. 3 e–f, the control and model groups were found to be significantly separated, with a good degree of intra-group clustering and significant differences between groups. And small molecule metabolites were objectively screened by embedded VIP and p value.

#### Differential lipid metabolites

A total of 426 differential lipid metabolites (277 lipid metabolites detected in positive ion mode and 149 in negative mode) were identified between model and control groups according to accurate m/z and MS/MS fragments. The detected differential lipid metabolites were from three major lipid types, including sphingomyelins, glycerolipids and fatty acyl groups. Based on the OPLS-DA model, 44 lipid metabolites with significant differences were screened according to VIP > 1 and *p* value < 0.05. These 44 differential metabolites contained 8 fatty acids (FA), 1 lysophosphatidyl ethanolamine (LPE), 11 plasmenylethanolamines (PE-O), 2 sphingomyelins (SM), 13 plasmenylphosphatidylcholines (PC-O), 1 ceramide (Cer), 2 diglycerides (DG), 2 triglycerides (TG) and 1 cholesterol (CE).

#### The regulation of BYHWD on lipid differential metabolites

Based on the 44 differential metabolites that existed between the model and control groups obtained above, these differential metabolites were analyzed by t test between the BYHWD, model and control groups. After the treatment with BYHWD, a total of 41 differential lipid metabolites were found to show significant regression (p < 0.01).

**Fig. 4:**
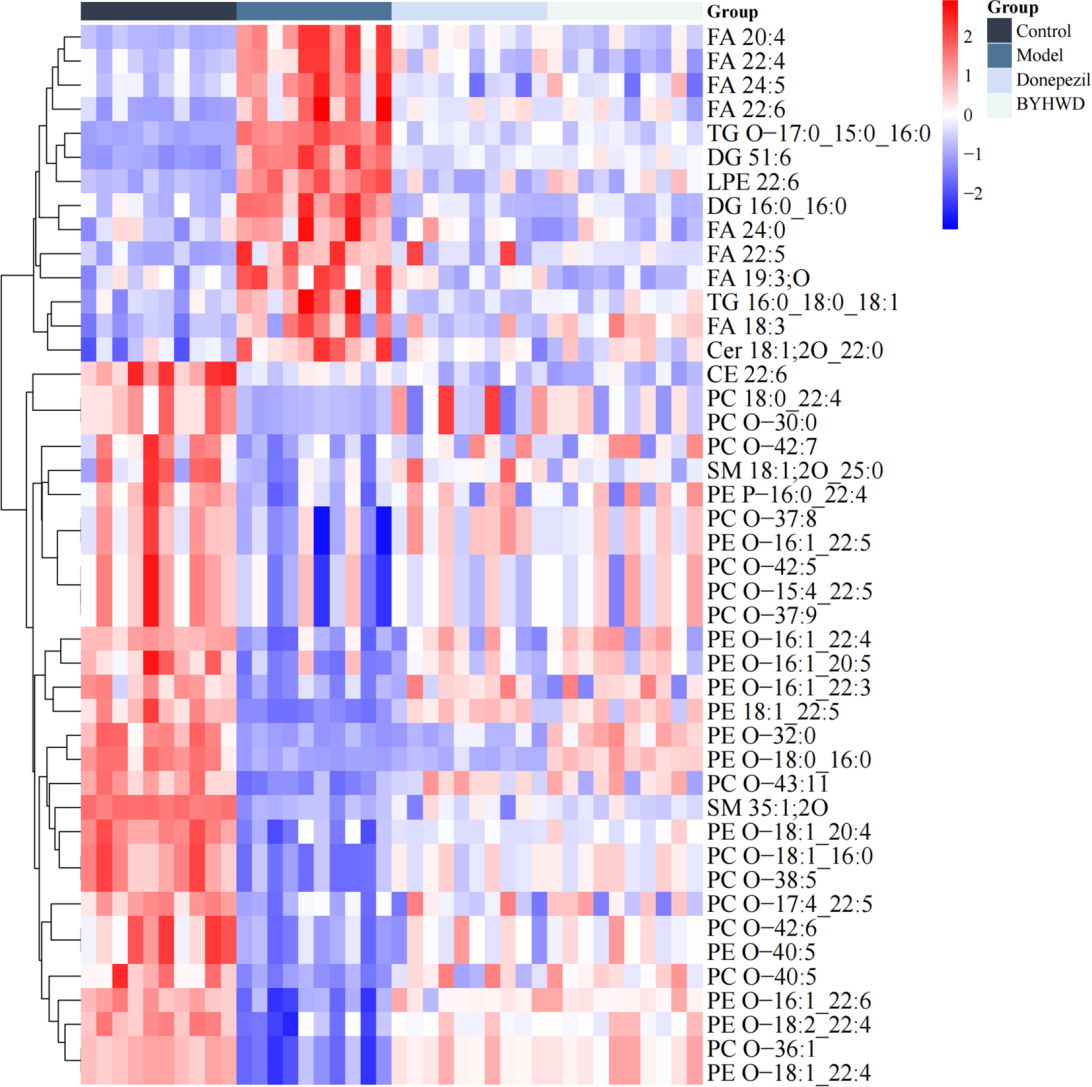
Heatmap of differential metabolites. Color changed from blue to red, corresponding to a progressive increase in concentration

Through screening and analysis of specific lipid species, it was found that three types of lipids had representative changes, which were very long-chain fatty acids (VLCFAs), plasmenylethanolamines (PE-Os, sn-1 position of phosphatidylethanolamine with ether bond) and plasmenylphosphatidylcholines (PC-Os, sn-1 position of phosphatidylcholine with ether bond) (Fig. 4).

VLCFAs, PE-Os, and PC-Os are essential during the developing process of AD. The levels of VLCFAs in blood, brain of AD patients are increased. Saturated fatty acids, especially VLCFAs (> 20C), can promote the production of Aβ through a variety of mechanisms, accelerating the pathological process of AD [5]. The levels of VLCFAs in the brain of rats increased, and obvious learning and memory disorders happened in rats, accompanied with the increase of Aβ levels [26]. Furthermore, the pathophysiological process of AD is closely related to phospholipids (PE-Os and PC-Os). Both PE-Os and PC-Os can inhibit the level of Aβ [27, 28].

**Fig. 5:**
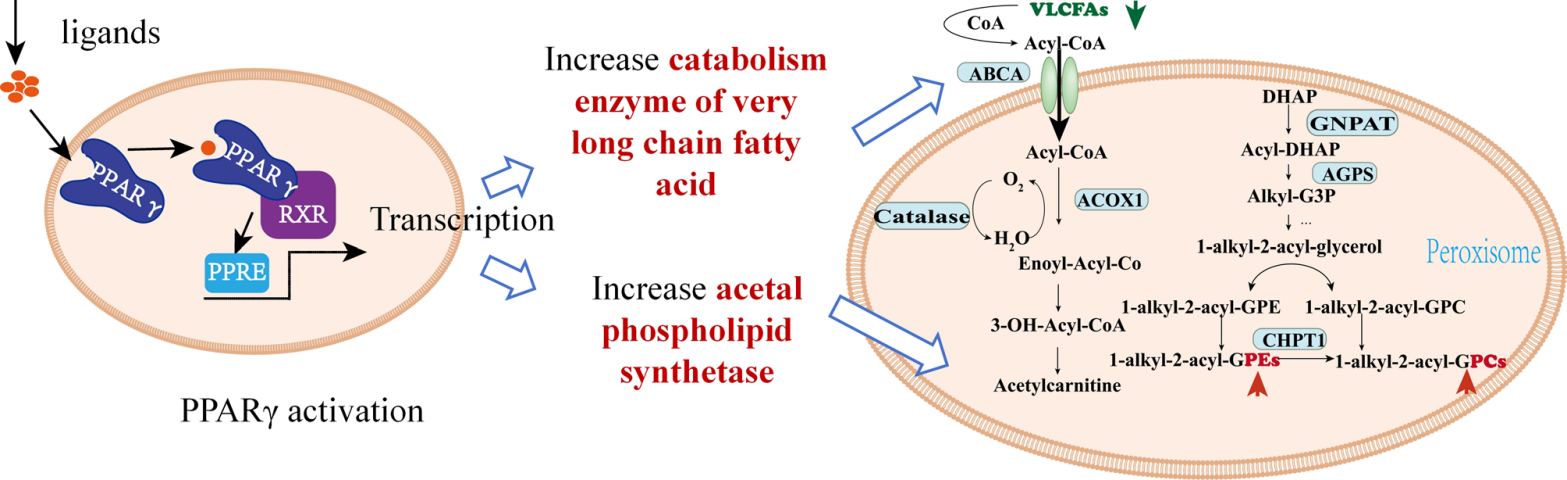
The role of PPARγ pathway in lipid regulation

The peroxisome proliferator-activated receptor-gamma (PPARγ) family is the key lipid-binding receptor to regulate the metabolism and balance of VLCFAs, PE and PC [29]. The VLCFAs catabolism, PEs anabolism, and PCs anabolism all happen in peroxisomes (Fig. 5). And PPARγ is a peroxisome proliferator-activated receptor. Once PPARγ is activated, more peroxisome will generate. A large number of literatures have reported that PPARγ play a key role in lipid metabolism disorder of AD [30].

The lipidomics results implied that the pivotal role of PPARγ in AD-related lipid metabolism, whereby its activation promotes peroxisome-mediated regulation. Consequently, it could be inferred that BYHWD exerts its therapeutic effect on AD through modulation of the PPARγ pathway.

### Constituents of BYHWD absorbed brain

**Fig. 6:**
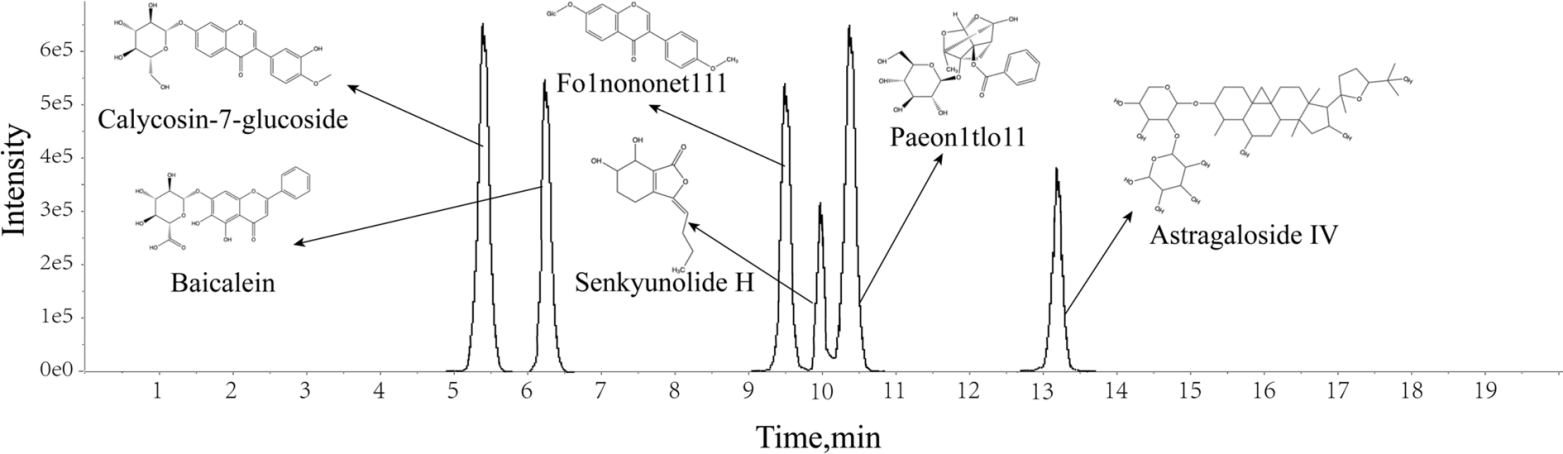
SIM mass chromatography of six constituents absorbed into the mice brain

In order to identify the key substances responsible for the anti-AD effects of BYHWD, we used UPLC-Q-TOF–MS analysis to detect the absorption components in hippocampal tissues of HFD & D-gal model mice after oral administration of BYHWD. The information on the chemical composition of BYHWD into hippocampal tissues is shown in Supplementary Material 2 Tab. S1. Compared with the MS spectrums of control mice, five original components (calycosin-7-glucoside, paeoniflorin, astragaloside IV, formononetin, senkyunolide H) and one metabolite (baicalein) were identified by UPLC-Q-TOF–MS in the mice hippocampal tissue treated with BYHWD (Fig. 6). These findings suggested that these six absorbed components might directly contributed to the pharmacological effects exerted by BYHWD on the brain.

According to the results of lipidomics study of BYHWD, PPARγ was revealed to be the potential target receptor. Whether the six absorbed constituents in hippocampal could regulate the lipid metabolism to therapy AD is the crucial aspect to be verified. Thus, a molecular docking technology was employed to analyze the binding ability of these six components with PPARγ.

### Molecular docking

**Fig. 7:**
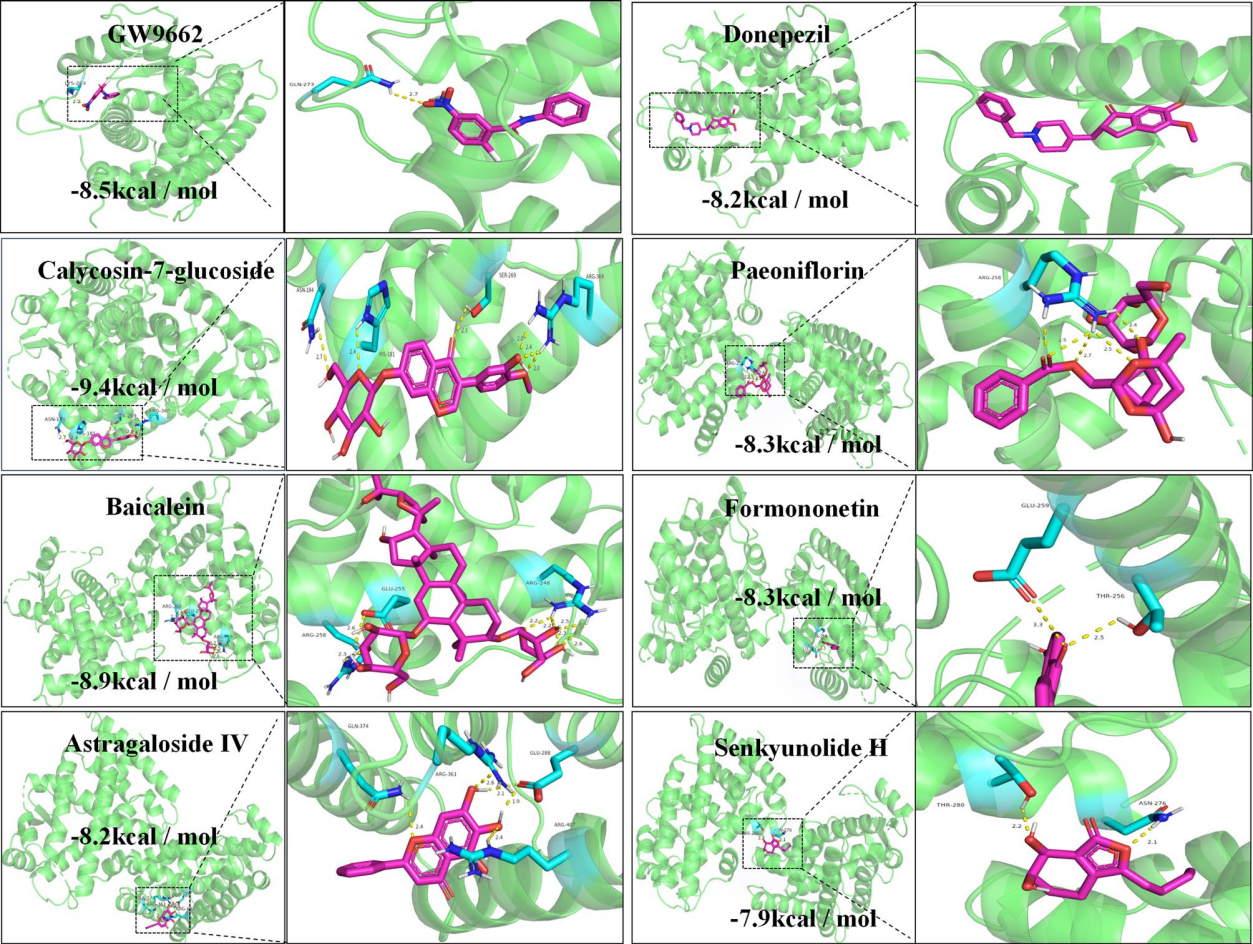
GW9662, donepezil and six brain-entry components of BYHWD (Calycosin-7-glucoside, Paeoniflorin, Baicalein, Formononetin, Astragaloside IV, Senkyunolide H) with PPARγ interaction diagram

Molecular docking analysis was conducted to investigate which component from the six brain components obtained through in vivo component analysis of BYHWD could bind to PPARγ. The binding energy (kcal / mol) reflects the affinity between small drug molecules and their target. Lower binding energy indicates more stable binding [31]. The values of binding energy of six components were utilized as an indicator. The results from molecular docking are presented in Fig. 7, revealing that CG exhibited the lowest binding energy (-9.4 kcal/mol) with PPARγ among all tested components, which implying that CG had excellent PPARγ binding activity. Therefore, CG was identified as the primary active ingredient responsible for exerting anti-AD effects within BYHWD.

### The effect of CG on PPARγ signaling pathway was verified in vitro

In vivo component analysis reveals that CG is the predominant brain-active constituent of *Scutellaria baicalensis* in Buyang Huanwu decoction. The chemical formula for CG is C_22_H_22_O_10_. This compound exhibits a broad spectrum of pharmacological activities, including immune system enhancement, antioxidant and anti-radiation properties, significant anticancer effects, and protective actions on the cardiovascular, cerebrovascular, hepatic, renal, and pulmonary systems. Furthermore, CG demonstrates neuroprotective effects, memory improvement, vascular smooth muscle relaxation, hormone-like activity, antibacterial and antiviral inhibition, lipid and glucose level reduction, and decreased incidence of diabetic complications [32–36]. In this study, six brain-penetrating components from Buyang Huanwu decoction were selected for molecular docking experiments with PPARγ. The results indicated that CG has a strong binding affinity with PPARγ, thereby being chosen as the primary focus of this research. Based on the aforementioned results, it is evident that CG serves as the primary active constituent responsible for exerting therapeutic effects in BYHWD. This effect is mediated through its binding affinity to PPAR receptors. Consequently, this study substantiated this effect via molecular biology investigations.

#### The cell viability of CG

**Fig. 8:**
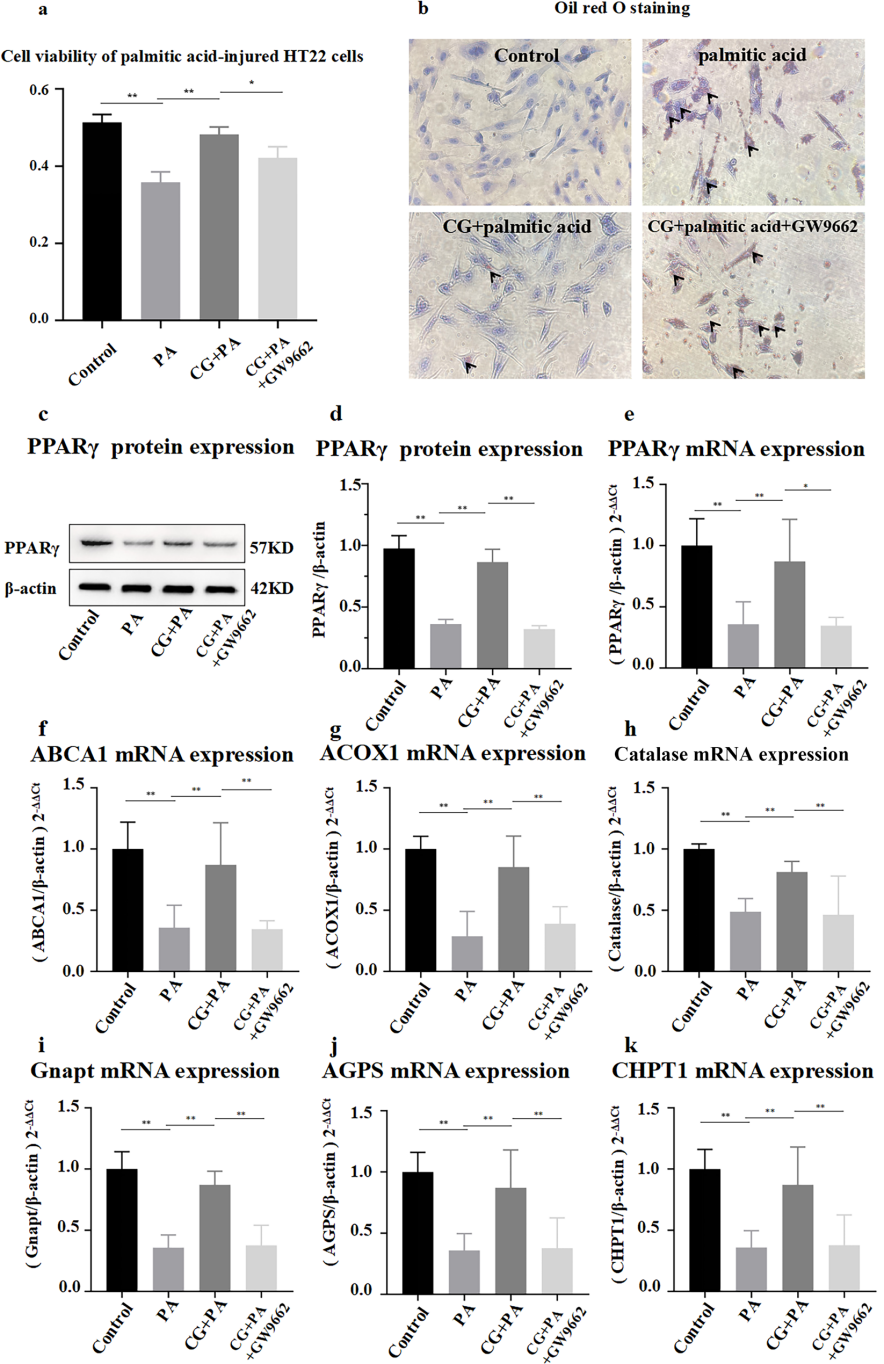
The effect of CG on PPARγ signaling pathway was verified in vitro.** a** Cell viability of palmitic acid (PA) injured HT22 cells treated with CG and PPARγ blocker GW9662; **b** HT22 cell Oil Red O staining (×200, The black arrows are grease drops); **c-d** Relative expression levels of PPARγ protein in cells of each group (mean ± SD, *n* = 3); **e** Relative expression levels of PPARγ mRNA; **f–h** Relative expression levels of ABCA1, ACOX1 and catalase mRNA; **i–k** Relative expression levels of GNPAT, AGPS and CHPT1 mRNA (mean ± SD, *n* = 3); Data are expressed as the mean ± SD. *P < 0.05, **P < 0.01

Palmitic acid is a saturated fatty acid that is lipotoxic to different types of cells [37]. To figure out the regulative effect of CG on AD in vitro, palmitic acid-injured HT22 cells were established by palmitic acid treatment. In order to further clarify the neuro protective mechanism of CG in AD models in vitro, PPARγ antagonist GW9662 was used for its neuro protective mechanism validating in this study. GW9662 is a selective PPAR antagonist that acts on PPARγ. GW9662 binds to Cys (285) on PPARγ. The cell proliferation activity of HT22 was determined by MTT assay which were treated by palmitic acid, CG and GW9662. By measuring the survival rate of HT22 cells with palmitic acid treatment before CG application, we investigated the effect of this component. As show in Fig. 8a, the cell viability was notably inhibited by palmitic acid compared to the control group (*p* < 0.01), while the cell proliferation inhibitory on HT22 was alleviated by CG treatment (*p* < 0.01). The cell proliferation rate of GW9662 was significantly lower than CG group (*p* < 0.01). These suggested that palmitic acid injury might lead to reduce lipid accumulation and cell viability in HT22 cells. The survival percentage of HT22 cells was increased after CG pretreatment, indicating that CG may be able to repair HT22 cells damaged by palmitic acid while GW9662 reversed the neuroprotective effect of CG. It suggested that the neuroprotective effect of CG may be related to PPARγ.

#### Oil Red O staining

Oil Red O staining was used to evaluate the lipid accumulation of CG and PPARγ receptor blocker GW9662 on palmitic acid-induced HT22 cells. Cell Oil Red O staining was mainly used to show the steatosis and abnormal lipid deposition of cells, and the positive staining results for the presence of fat droplets in cells were orange to red [38]. As shown in Fig. 8b, a large number of orange fat droplets appeared in HT22 cells induced by palmitic acid. The HT22 cells treated with palmitic acid exhibited a significant increase in the number of cells stained with Oil Red, accompanied by the formation of clearly visible lipid droplets, when compared to the control group. Fewer Oil Red colored HT22 cells existed in the group treated with CG compared to palmitic acid, and no clearly visible orange lipid droplet formations could be observed in the cells. Furthermore, we found that cells treated with GW9662 had significantly increase lipid accumulation compared with cells treated with CG.

These results suggested that CG could effectively improve this phenomenon which could be inhibited by PPARγ blocker GW9662. The above results vindicated that CG ameliorated palmitate induced lipid accumulation in HT22 cells by activating PPARγ.

#### PPARγ expression

Integrating hippocampal biochemistry, lipidomics results and molecular docking analysis, the VLCFA catabolism as well as plasmalogen anabolism, PPARγ pathway involved in the effect of CG. Meanwhile, accumulated evidence has shown that activation of PPARγ pathway could induce peroxisome proliferation, and then affect VLCFA decompose and plasmalogen synthesis, which was an important mechanism of AD occurrence and development [39]. Therefore, western blotting and RT-PCR were used to test and verify the effect of CG on PPARγ signaling pathway.

To further determined whether CG played a role in regulating lipid metabolism disorder by activating PPARγ receptor, we detected the expression of PPARγ protein and mRNA in cells of each group using western blotting and RT-PCR. As we can see from the western blotting result (Fig. 8 c-d), CG could reverse markable decrease of PPARγ protein expression in HT22 cells.

The protein and mRNA expressions of PPARγ were decreased in palmitic acid-induced model group, whereas these reduced protein and mRNA levels were significantly increased after the treatment with CG. Interestingly, however, PPARγ protein and mRNA expression levels were significantly reversed in the PPARγ blocker GW9662 group (*p* < 0.01) (Fig. 8 e). These results demonstrated that the potential mechanism of CG to alleviate AD might correlate with the activation of PPARγ receptor pathway.

#### mRNA expression of long-chain fatty acid catabolic metabolizing enzymes

The aforementioned findings suggest that CG may exert its anti-AD effects through the PPARγ pathway, given that peroxisomes serve as the primary site for lipid metabolism, including long-chain fatty acid metabolism and phosphatidylcholine synthesis. Combining lipidomics analysis results, it was shown that VLCFAs accumulated and phosphatidylserine was greatly reduced in the AD model group, so it was hypothesized that PPARγ may promote peroxisome proliferation, increase the decomposition of VLCFAs and the synthesis of phosphatidylserine (PEs and POs), thereby alleviating long-chain fatty acid accumulation and increasing the level of phosphatidylserine in the brain, thereby improving the cognitive function of AD. Therefore, we proceeded to quantify the mRNA expression levels of pivotal enzymes involved in the metabolism of VLCFAs degradation (ABCA1, ACOX1, and Catalase) as well as glycerophospholipid synthesis (GNPAT, AGPS, and CHPT1) using the RT-PCR technique.

The expression levels of mRNA of ABCA1, ACOX1 and catalase in the cells of other groups were significantly reduced compared with the controls (*p* < 0.01). After CG treatment, the expression levels of ABCA1, ACOX1 and Catalase were upregulated (*p* < 0.01). Moreover, GW9662 inhibited the expression of ABCA1, ACOX1 and Catalase (*p* < 0.01) (Fig. 8 f–h). The above results demonstrated that CG could enhance the catabolic metabolism of VLCFAs. However, the activity of CG is impeded by PPARγ inhibitors, suggesting that CG activated the PPARγ pathway to upregulate intracellular catabolic metabolism of long-chain fatty acids, thereby ameliorating their accumulation in the brain. To further investigate whether CG regulates sphingomyelin synthesis metabolism through the PPARγ pathway, we assessed mRNA expression levels of key enzymes involved in sphingomyelin synthesis metabolism.

#### mRNA expression of the plasmalogen synthesizing enzymes

The reduction of plasmalogen levels in the brain of AD patients was a typical pathological change in AD [40], which was consistent with the results of this study. Because plasmalogen synthesis metabolism occurs in the peroxisome, and PPAR is a peroxisome proliferator receptor, the mRNA expression of key enzymes involved in plasmalogen synthesis metabolism was measured. The expression levels of mRNA content of GNPAT, AGPS and CHPT1 in the cells of other groups were significantly reduced compared with the controls (*p* < 0.01). After CG treatment, the expression levels were upregulated (*p* < 0.01). Moreover, GW9662 also inhibited the expression of GNPAT, AGPS and CHPT1 (*p* < 0.01) (Fig. 8 i-k). The upregulation of GNPAT, AGPS, and CHPT1 mRNA expression by CG was attenuated by a PPARγ inhibitor, implying that CG enhances sphingomyelin synthesis via the PPARγ pathway. In summary, CG can promote the catabolism of long-chain fatty acids and also upregulate the synthesis of plasmalogen. The findings suggested that CG modulated lipid metabolism through the activation of PPARγ receptor, facilitating peroxisome proliferation and enhancing lipid metabolism within peroxisomes. Specifically, it promotes the breakdown of long-chain fatty acids and the synthesis of phosphatides, thereby stabilizing lipid metabolism in the brain of AD mice and restoring cognitive function.

## Discussion

Both aging and obesity are associated with an elevated risk of diabetes, vascular disease, hypertension, dyslipidemia, and dementia [41, 42]. To investigate the pathophysiology of aging, D-galactose (D-gal) has been utilized in induced aging models, which exhibit findings comparable to those observed in natural aging models [43, 44]. Previous studies have demonstrated that D-gal-induced aging results in cognitive decline and brain lesions, including microglial activation, synaptic dysfunction, increased reactive oxygen species production, apoptosis, and amyloid deposition [45–47]. In addition to aging, obesity is an independent risk factor for the development of dementia [5]. Chronic consumption of a high-fat diet (HFD) is a primary cause of obesity [48]. Studies have shown that prolonged HFD consumption can lead to obesity-related insulin resistance and brain lesions, such as increased oxidative stress, microglial activation, autophagy impairment, apoptosis, synaptic dysfunction, and enhanced amyloid deposition, ultimately resulting in cognitive decline [49–52]. Furthermore, obesity exacerbates blood–brain barrier disruption, neuroinflammation, oxidative stress, and gene dysfunction related to synaptic function in older rats [53–55]. Shwe T et al. investigated HFD-fed rats treated with subcutaneous D-gal injections, revealing decreased cognitive function, impaired hippocampal autophagy, abnormal synaptic protein expression, increased amyloid-producing enzyme activity, heightened oxidative stress, microglial malformation, apoptosis, and reduced dendritic spine density. Moreover, compared to rats treated solely with HFD or D-gal, those subjected to HFD combined with D-gal exhibited higher levels of oxidative stress, apoptosis, and dendritic spine loss [56].

These findings suggest that the HFD combined with G-gal model exhibits characteristics similar to those observed in clinical AD patients, making it suitable for constructing an AD model. Although these studies did not directly demonstrate increased amyloid-beta (Aβ), rats subjected to HFD combined with G-gal exhibited elevated expression of the amyloid-producing enzyme BACE1 in the brain and an increased proportion of SA-β-gal positive cells in the CA3 region of the hippocampus. Aβ is generated through the sequential cleavage of amyloid precursor protein (APP) by BACE1, and elevated BACE1 activity has been detected in human AD brain extracts, consistent with experimental evidence showing that neurons in AD produce higher levels of Aβ compared to normal aging. Our results also indicate hyperphosphorylation of Tau in the hippocampus of the HFD combined with G-gal model. During AD progression, the production of Aβ and the hyperphosphorylation of Tau are interdependent processes [57, 58], both representing classic pathological changes and hallmark features of AD.

AD is a late-onset neurodegenerative disorder characterized by the presence of intracellular neurofibrillary tangles composed of hyperphosphorylated microtubule-associated protein Tau and extracellular Aβ plaques derived from amyloid precursor proteins. Dysregulation of brain lipids represents one of the key hallmarks of Alzheimer's disease [59], and while circulating cholesterol has been the most extensively studied lipid in this context [60], emerging evidence highlights the significance of other lipids, such as phospholipids, in AD pathogenesis.

Plasmalogens, a subclass of glycerophospholipids characterized by vinyl ether bonds at the sn1 position of the glycerol backbone [17, 28, 61–65], are integral components of cellular membranes. They play a crucial role in the pathophysiology of AD by interacting with cholesterol to modulate membrane fluidity and lipid microdomain composition. Plasmalogens enhance the activity of α-secretase, leading to the non-amyloidogenic cleavage of amyloid precursor protein (APP) into non-amyloidogenic peptides, thereby reducing the formation of amyloid plaques, a hallmark of AD neuropathology [66]. Age-related declines in circulating plasmalogen levels may impair phosphatide utilization by the central nervous system, potentially increasing the risk of AD. Kling MA et al. [67] employed lipidomics approaches to quantify changes in four ethanolamine plasmalogens (PlsEtns). Their findings suggest that alterations in PlsEtn biosynthesis and/or remodeling in the serum of AD patients may be associated with the progression of Tau and amyloid pathology. These results align with previous studies [65, 68, 69].

Phosphatidylcholine (PC), a type of phospholipid predominantly located in the cell membranes of neurons, plays a crucial role in regulating cell signaling, protein trafficking, and energy metabolism within the brain [70, 71]. In AD patients, PC levels are markedly reduced in the frontal, primary auditory, and parietal cortices, while their metabolites are elevated [72–74]. Previous studies have also reported that certain ether-linked PC species, such as PCO-36:1, are associated with Tau measurements or tau/Aβ_42_ ratios in cerebrospinal fluid [75–77]. Very long-chain fatty acids (VLCFAs) are essential for maintaining membrane stability, modulating cell signaling, and regulating inflammation. Kou J et al.[78] observed in postmortem AD brains that VLCFAs accumulated in cortical regions linked to AD neuropathology, whereas phosphatides in the medial frontal gyrus decreased. Pena-Bautista C et al. [79] performed epigenomic and lipomic analyses on plasma samples from patients with mild cognitive impairment due to AD and healthy controls. Their findings identified phosphatidylethanolamine (PE), lysophosphatidylcholine (LPC), ceramides, phosphatidylcholine (PC), triglycerides (TG), and several long-chain fatty acid families as differentially expressed in AD.

Over the past few decades, peroxisomes and their physiological significance in health and disease have garnered considerable attention. As an organelle present in nearly all eukaryotic cells, peroxisomes, along with mitochondria, form a critical metabolic platform for regulating various fatty acid oxidation processes and redox reactions, playing a pivotal role in lipid metabolism [80]. The peroxisome is instrumental in lipid biosynthesis, particularly as the primary site for the synthesis of alkyl (ether) lipids [81]. DHAP acyltransferase catalyzes the first step in ether lipid biosynthesis, occurring on the lumenal side of the peroxisomal membrane. Subsequent synthesis involves enzymes within the peroxisomal matrix, including glycerophospho-o-acyltransferase (GNPAT) and alkylglycerone phosphate synthase (AGPS), which are crucial for the production of phosphatidylethanolamine (PE) and phosphatidylcholine (PC) [82]. Additionally, peroxisomes are responsible for the regulation of long-chain and very-long-chain fatty acyl-CoA shortening, dicarboxylic fatty acids, 2-methyl-branched fatty acids, arachidonate-derived inflammatory mediators, prostaglandins, and bile acid intermediates. Very-long-chain fatty acids (VLCFAs) and branched-chain fatty acids undergo β-oxidation primarily within peroxisomes [83]. Functional peroxisomes are essential for cellular metabolism, and deficiencies in peroxisomal enzymes can lead to significant abnormalities in brain and organ function. Kou J et al. [84] observed a decrease in acetal phospholipids and an increase in VLCFA and peroxisome volume density in postmortem AD patient brains, suggesting substantial peroxisome-related changes that may contribute to the progression of AD pathology.

PPARγ is a prominent lipid-activated nuclear receptor (NR) involved in adipocyte differentiation. It binds to thousands of genomic sites, including numerous genes associated with glucose and lipid metabolism, typically forming a heterodimer with RXR [85]. Our experimental findings suggest that the anti-AD effects of BTHWD may be linked to PPARγ-mediated regulation of VLCFA degradation, glycerophospholipid synthesis, as well as the expression of fatty acid (FA)-related and glycerophospholipid-related genes, which exhibit synchronous changes with PPARγ gene expression. Although the interactions between PPARγ and these genes have not been fully elucidated, several studies have reported similar observations.

PPARγ and ABCA1: ABCA1 is a key downstream target gene of PPARγ. Studies have demonstrated that activation of PPARγ1 and LXRα by natural or synthetic ligands leads to the transactivation of ABCA1, ABCG1, and ApoE, thereby promoting cholesterol efflux from foam cells [86].

PPARγ and ACOX1: Wang Y et al. found that high-intensity interval training (HIIT) increases mRNA levels of fatty acid oxidation-related genes (PPARα, CPT1α, and ACOX1) while decreasing mRNA levels of lipogenesis-related genes (PPARγ), thus improving liver metabolism in type 2 diabetic mice [87]. This result reflects the concurrent changes in PPARγ and ACOX1 gene expression in mice, consistent with our findings.

PPARγ and Catalase: Catalase gene expression is regulated by various mechanisms, including PPARγ, TNF-α, p53 protein, and CpG island methylation in the promoter region [88]. PPARγ and Catalase control CAT gene expression by binding to a distal PPARγ response element (PPRE) in the gene promoter region [89, 90].

PPARγ and CHPT1: Du S et al. Integration of network pharmacology, lipidomics, and transcriptomics analysis to reveal the mechanisms underlying the amelioration of AKT-induced nonalcoholic fatty liver disease by total flavonoids in vine tea. They discovered that the CHPT1 gene and PPARγ protein levels changed synchronously [91], which is similar to our results.

The peroxisome proliferator-activated receptor (PPAR) family is a group of intracellular lipid-binding receptors that function as central regulators of peroxidation and β-oxidation processes [92]. As a member of the nuclear hormone receptor superfamily, PPARγ exhibits a classical domain structure characteristic of this family. Specifically, PPARγ comprises an N-terminal A/B region containing a ligand-independent transactivation subdomain (AF-1), a DNA-binding domain (DBD) with two zinc finger motifs, a ligand-binding domain (LBD), and a C-terminal ligand-dependent transactivation domain (AF-2). The LBD of PPARγ selectively binds to both endogenous and exogenous ligands [93]. Upon activation by these ligands, PPARγ forms a heterodimer with retinoid X receptor (RXR) [94], which subsequently translocates into the nucleus and binds to the PPRE enhancer region via its DBD. This interaction enhances the transcriptional activity of PPARγ, initiating downstream metabolic pathways, such as fatty acid metabolism and glycerophospholipid metabolism [95]. Recent research has highlighted the therapeutic potential of PPARγ agonists in neurodegenerative diseases, including cerebral ischemia/brain injury, AD, and Parkinson's disease (PD) [96–98]. However, studies have also reported adverse events associated with PPARγ agonist therapy, such as peripheral edema [99]. Whether targeting PPARγ alone can fully address the multifaceted mechanisms underlying neurological disorders remains to be determined.

Donepezil, a piperidine-based non-competitive and reversible inhibitor, is one of the five approved therapeutic drugs for ADand is widely prescribed as a first-line treatment for patients with mild to moderate AD. Previous studies have indicated that donepezil's pharmacological effects may be linked to its role in regulating lipid metabolism abnormalities associated with AD. Genes related to lipid metabolism, such as APOE, CYP46, CETP, and ABCA1, have been identified as potential pharmacodynamic markers for evaluating donepezil's efficacy [100–104]. However, the clinical application of donepezil is significantly constrained by its severe adverse reactions, which are often attributed to cholinergic effects on the gastrointestinal and nervous systems. Common side effects include muscle fatigue, cramps, bradycardia, nausea, diarrhea, dizziness, and insomnia. More severe symptoms can involve convulsions, urinary incontinence, and rashes.

BYHWD is a traditional Chinese medicine formula that has been transmitted through millennia. It is meticulously composed of precise proportions of each ingredient and has historically been utilized to treat vascular dementia, Alzheimer's disease, and other related conditions, demonstrating significant overall efficacy. Compared to donepezil or PPARγ agonists, BYHWD contains a more complex composition with multiple targets, resulting in fewer side effects. This allows for a more comprehensive intervention from various perspectives in the treatment of Alzheimer's disease and other multi-mechanism diseases. CG, an active component derived from Astragalus membranaceus, a natural plant constituent of BYHWD, was the focus of this study to elucidate its anti-AD mechanism via PPARγ. Notably, BYHWD also comprises numerous other neuroprotective pharmacological substances that synergistically contribute to its potent anti-AD functions. Further exploration of these mechanisms will be conducted in subsequent studies.

## Conclusion

In this study, it was observed that BYHWD effectively ameliorated the cognitive and memory deficits in a HFD & D-gal mice model of AD as evidenced by improvements in behavioral and histopathological pharmacodynamic indices. In vivo component analysis revealed the presence of six components of BYHWD in the brain. Lipidomics and molecular interlinking analyses indicated that the main pharmacodynamic substance, CG, present in BYHWD might activate the PPARγ pathway to induce peroxisome proliferation and regulate lipid metabolism disorders in the AD mice brain, thereby achieving therapeutic effects against AD. Furthermore, an in vitro experiment using palmitic acid-induced HT22 cells confirmed these findings and provided insights into the mechanism underlying BYHWD's treatment of AD from a lipid metabolism perspective.

## Supplementary Information

Below is the link to the electronic supplementary material.

Supplementary file1 (DOC 12388 KB)

Supplementary file2 (XLSX 17 KB)

## Abbreviations

BYHWD: Buyang Huanwu Decoction
BHD-H: Buyang Huanwu Decoction high dosed
BHD-M: Buyang Huanwu Decoction middle-dosed
BHD-L: Buyang Huanwu Decoction low-dosed
HFD & D-gal: High-fat diet and D-galactose
P-tau: Hyperphosphorylated tau protein
AD: Alzheimer's disease
CG: Calycosin-7-glucoside
Aβ: Amyloid-beta
HDLC: High-density lipoprotein
LDLC: Low-density lipoprotein
TG: Triglyceride
TC: Total cholesterol
MWM: Morris water maze
H&E: Hematoxylin & eosin staining
ABCA1: ATP-binding cassette transporter A1
Catalase: Catalyzes the breakdown of hydrogen peroxide
ACOX1: Acyl-CoA oxidase 1
DHAP: Dihydroxyacetone phosphate
GNPAT: Glyceronephosphate O-acyltransferase
AGPS: Alkylglycerone phosphate synthase
G3P: 1–0-Alkyl-2-hydroxy-sn-glycerophosphate
GPEs: 1–0-Alkyl-2-acyl-sn-GPEtn
GPCs: 1–0-Alkyl-2-acyl-sn-GPCtn
CHPT1: Choline phosphotransferase 1
IHC: Immunohistochemistry
PE-O_S_: Plasmenylethanolamines
PC-Os: Plasmenylphosphatidylcholines
PPARγ: Peroxisome proliferator-activated receptor-gamma
VLCFAs: Very long-chain fatty acids
PA: Palmitic acid
WB: Western blotting
RT-PCR: Reverse transcription-polymerase chain reaction
