# Supplementary material for: Investigation of the mechanism of Buyang Huanwu decoction in improving learning and memory impairment in Alzheimer's disease mice based on lipidomics
Source: J Nat Med. 2025 Apr 7;79(3):568–90. doi: 10.1007/s11418-025-01890-x (PMC12058831; doi:10.1007/s11418-025-01890-x)
Supplement: Supplementary file 1 — Supplementary file1 (DOC 12388 KB) [file 11418_2025_1890_MOESM1_ESM.doc]

**Investigation of the Mechanism of Buyang Huawan Decoction in Improving Learning and Memory Impairment in** **Alzheimer's disease Mice Based on Lipidomics**

**Jing Jiang^1,2^, Kai Duo^2^, Siyu Zhu^1^,** **Yitong** **Wang^2^, Hui Xue****^1^, Chengyu Piao^1^,** **Yifan Ren^1^, Xia Lei^3^,** **Yafeng Zhang^3*^, Jianxin Liu^4^,** **Lihong Yang****^2*^, Ning Zhang^1*^**

^1^College of Pharmacy, Heilongjiang University of Chinese Medicine, Harbin, Heilongjiang, China,

^2^Heilongjiang Institute for Drug Control, Harbin, Heilongjiang, China,

^3^Jiangsu CM Clinical Innovation Center of Degenerative Bone & Joint Disease, Wuxi TCM Hospital Affiliated to Nanjing University of Chinese Medicine, Wuxi, Jiangsu, China,

^4^School of Pharmaceutical Sciences, China-Pakistan International Science and Technology Innovation Cooperation Base for Ethnic Medicine Development in Hunan Province, Hunan University of Medicine, Huaihua, Hunan, China

*** Correspondence:**

Ning Zhang; [zhangning0454@163.com](mailto:zhangning0454@163.com)

Lihong Yang; [bjsnowman@126.com](mailto:bjsnowman@126.com)

Yafeng Zhang; [wxzy007@njucm.edu.cn](mailto:wxzy007@njucm.edu.cn)


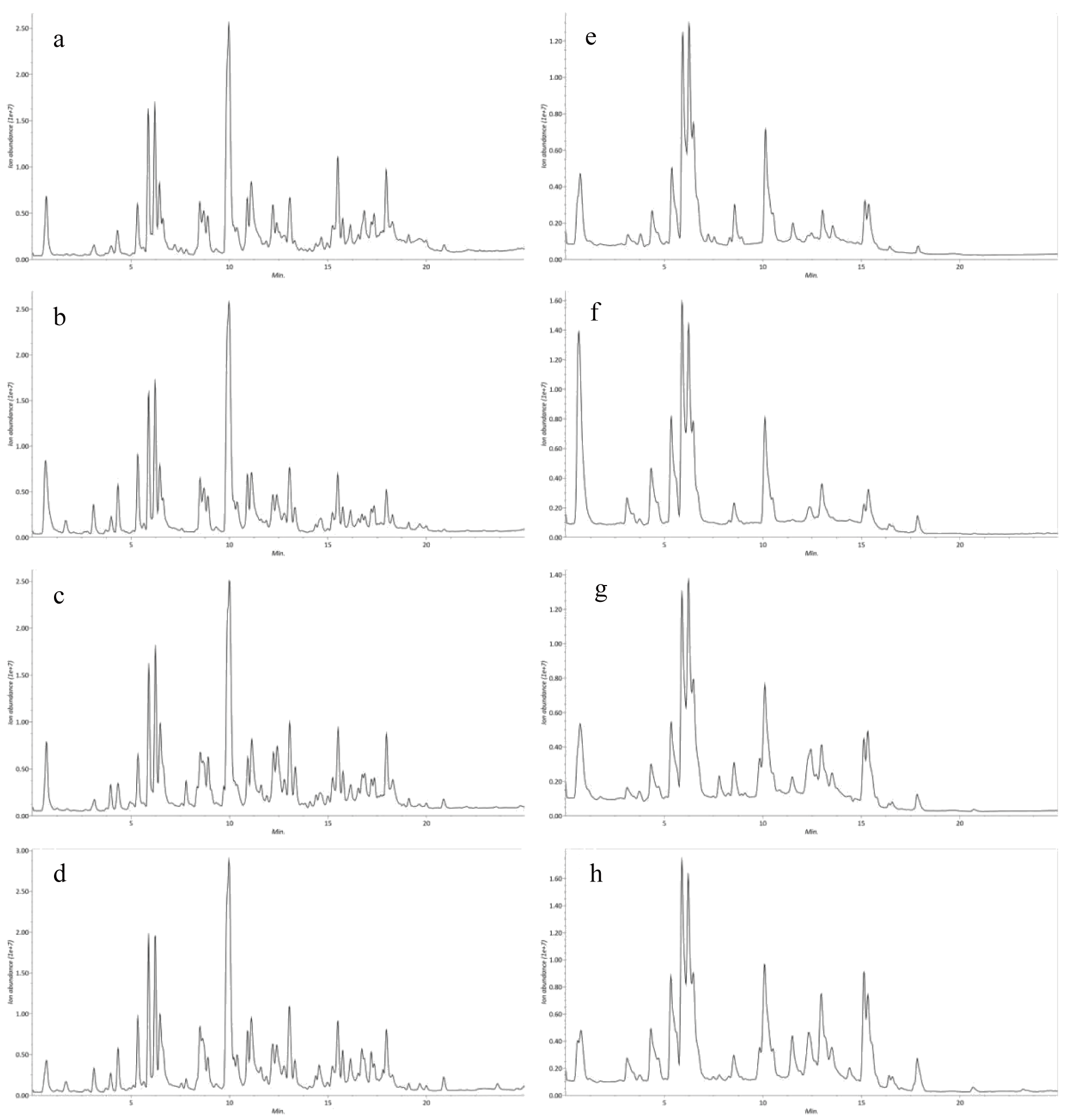


Figure S1 Total ions chromatogram of hippocampus lipidomics in positive and negative ion mode. Positive ion mode, (**a**) Control group, (**b**) Model group, (**c**) Donepezil group, (**d**) BYHWD group; Negative ion mode, (**e**) Control group, (**f**) Model group, **(g)** Donepezil group, **(h)** BYHWD group


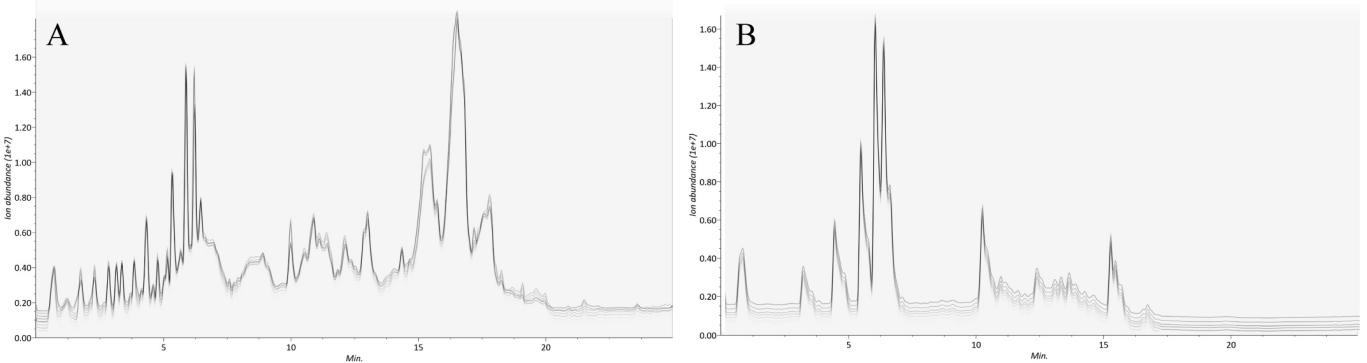


a

b

Figure S2 Total ions chromatogram of QC samples in positive and negative ion mode. **(a)** Positive ion mode, **(b)** Negative ion mode
